# Wolf’s Physiologic Chemistry

**Published:** 1902-04

**Authors:** 


### Books and Magazines.

Wolf's Physiologic Chemistry.—A laboratory hand-book of physiologic chemistry and urine-examination. By Charles G. L. Wolf, M. D., Instructor in Physiologic Chemistry, Cornell University Medical College, New York. 12mo volume of 190 pages, fully illustrated. Philadelphia and London: W. B. Saunders & Company. 1901. Cloth, $1.25 net.

This little volume is well adapted to serve as a practical laboratory guide and as a manual to meet the needs of undergraduate students and physicians making preparation for State board examinations. It is conveniently arranged and well written. The best approved methods of examination of the urine and stomach contents are so well described that they may be easily understood.

